# Development and Validation of a Severity Scale for Leprosy Type 1 Reactions

**DOI:** 10.1371/journal.pntd.0000351

**Published:** 2008-12-23

**Authors:** Stephen L. Walker, Peter G. Nicholls, C. Ruth Butlin, Jose Augusto C. Nery, Hemanto K. Roy, Emanuel Rangel, Anna M. Sales, Diana N. J. Lockwood

**Affiliations:** 1 Department of Infectious and Tropical Diseases, London School of Hygiene and Tropical Medicine, London, United Kingdom; 2 School of Health Sciences, University of Southampton, United Kingdom; 3 DBLM Hospital, Nilphamari, Bangladesh; 4 Oswaldo Cruz Institute, Rio de Janeiro, Brazil; Hospital Universitário, Brazil

## Abstract

**Objectives:**

To develop a valid and reliable quantitative measure of leprosy Type 1 reactions.

**Methods:**

A scale was developed from previous scales which had not been validated. The face and content validity were assessed following consultation with recognised experts in the field. The construct validity was determined by applying the scale to patients in Bangladesh and Brazil who had been diagnosed with leprosy Type 1 reaction. An expert categorized each patient's reaction as mild or moderate or severe. Another worker applied the scale. This was done independently. In a subsequent stage of the study the agreement between two observers was assessed.

**Results:**

The scale had good internal consistency demonstrated by a Cronbach's alpha >0.8. Removal of three items from the original scale resulted in better discrimination between disease severity categories. Cut off points for Type 1 reaction severities were determined using Receiver Operating Characteristic curves. A mild Type 1 reaction is characterized using the final scale by a score of 4 or less. A moderate reaction is a score of between 4.5 and 8.5. A severe reaction is a score of 9 or more.

**Conclusions:**

We have developed a valid and reliable tool for quantifying leprosy Type 1 reaction severity and believe this will be a useful tool in research of this condition, in observational and intervention studies, and in the comparison of clinical and laboratory parameters.

## Author Summary

Leprosy is caused by a bacterium and is curable with a combination of antibiotics known as multi-drug therapy which patients take for six or 12 months. However, a significant proportion of leprosy patients experience inflammation in their skin and/or nerves which may occur even after successful completion of multi-drug therapy. These episodes of inflammation are called leprosy Type 1 reactions. Type 1 reactions are an important complication of leprosy because they may result in nerve damage which leads to disability and deformity. Type 1 reactions require treatment with immunosuppressive agents such as corticosteroids. The severity of Type 1 reactions varies with time, treatment and between individuals. We have developed a clinical severity scale to measure the severity of Type 1 reactions. The scale has three sections. The first measures the involvement of the skin using the number of affected skin lesions, the degree of inflammation of those lesions and the presence of swelling of the hands, feet or face. The second section is a measurement of the sensory function of the nerves supplying the eyes, hands and feet by assessing a patient's ability to feel graded nylon fibres. The third section uses a standard measure of muscle power to assess motor function of the nerves of the face, hands and feet. The clinical severity scale we have developed will facilitate the study of Type 1 reactions and enable direct comparison between different studies. This will improve the management of this disabling complication of leprosy.

## Introduction

Leprosy is a chronic granulomatous disease caused by *Mycobacterium leprae*. More than 254 000 new cases were reported to the World Health Organization in 2007 [1].

The disease predominantly affects the skin and nerves. The nerve involvement associated with the disease may lead to permanent deformity and disability. A spectrum of disease phenotypes is recognised and these are determined by the host response to the organism [2]. The tuberculoid pole of the spectrum is characterised by strong host cell mediated immunity to the organism, whereas patients with lepromatous leprosy have a predominantly humoral immune response [3]. The borderline states of the disease are immunologically unstable.

Leprosy may be exacerbated by immunological complications–Type 1 (reversal) reactions and erythema nodosum leprosum (Type 2 reactions).

Type 1 reactions occur predominantly in individuals with the borderline forms of leprosy. They are characterised by inflammation of the skin, nerves or both. Type 1 reactions may occur before, during or after the successful completion of multi-drug therapy. Type 1 reactions affecting the peripheral nerves may result in decreased sensory and motor function and lead to disability. 20–30% of individuals diagnosed with leprosy will have a Type 1 reaction [4],[5].

Type 1 reactions are usually treated with oral corticosteroids but approximately 40% of individuals do not experience complete recovery of clinically detectable nerve function impairment (NFI) [6]. Clinical trials with appropriate outcome measures are needed to determine the most effective treatment regimens [7]. It has proved difficult to compare the small number of studies because of the different outcome measures used. There are also difficulties in comparing the severity of Type 1 reactions between different cohorts and even between different arms of clinical trials.

A tool which enables clinicians to accurately assess the severity of leprosy Type 1 reactions would be useful in defining outcomes for clinical trials. It would facilitate the even distribution of patients with similar disease severity between the arms of clinical trials. A measure of reaction severity could also be used in treatment guidelines to indicate the need for therapy. A quantitative measure of reaction severity may be a useful prognostic tool.

A scale devised as part of the ILEP Nerve Function Impairment and Reaction (INFIR) Cohort study examined 21 parameters for the basis of a severity scale of both Types of reactions and retrospectively assessed the performance of this scale [8]. There was good agreement between items in the scale.

A different scale (with 24 parameters) was used by Marlowe et al in a different INFIR study of azathioprine and prednisolone in Type 1 reactions but it was not validated [9]. An “indice névritique”–a composite scale using various assessments of nerves including electrophysiological studies–was developed by Naafs and colleagues but has not been validated [10],[11].

Using the INFIR scales as a starting point we decided to develop and validate a scale for Type 1 reactions and nerve function impairment in leprosy.

## Methods

### Expert opinion

A questionnaire was sent to eight leprologists who were not involved in the development of the current scale. The questionnaire used open questions to ascertain the signs they believed to be important in Type 1 reaction, which signs indicated a more severe reaction and how they categorised Type 1 reaction severity.

### Scale development

The severity scale for leprosy Type 1 reactions was developed by modifying the two previous scales used in the INFIR studies. The scale we developed and tested has 24 parameters grouped into three parts (see Appendix S1):

Section A contained six parameters which scored between 0 and 3 depending on the assessment of their severity by the examiner using the scale.

Section B is an assessment of sensory function of each of the trigeminal, ulnar, median and posterior tibial nerves. Cotton wool is used to assess the trigeminal nerve. Graded Semmes-Weinstein monofilaments (SWM) are used for the ulnar, median and posterior tibial nerves.

**Figure 1:**
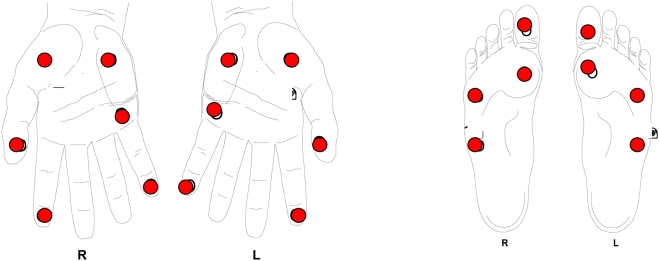
Test sites on the hands (2 and 10g) and the feet (10 and 300g).

The ulnar and median nerves are examined using a 2 and 10g monofilament at three sites on the palmar aspect of the hand for each nerve (ulnar and median) and the posterior tibial nerves are assessed using 10 and 300g at four sites on the sole of the foot (Fig. 1). A score from 0 to 6 was assigned depending on the ability of the patient to successfully recognise the weighted monofilaments and the number of sites in which they were felt. For example, on the hand if a person could feel the 2g monofilament at the three sites innervated by the ulnar nerve then a score of zero was recorded. If the 2g was felt at two sites and the 10g at the third site a score of one was recorded. If however the 10g monofilament was not felt at one site then a score of 4 was recorded even if the patient was able to feel the 2g monofilament at the other two sites.

Section C measures motor function of ten nerves (facial, ulnar, median, radial, posterior tibial) by voluntary muscle testing (VMT) using the MRC grading system [12]. Normal muscle power (MRC Grade 5) scores zero on the scale. Grade 4 scores 1 and grade 3 scores two. An MRC grade of less than three scores three on the severity scale.

The sum of the total for each section gives the overall severity scale score which ranges from 0–96, the lower the score the less severe the reaction.

### Scale testing

The assessment of the severity scale was performed at the specialist leprosy referral centres of DBLM Hospital, Nilphamari, Bangladesh and Oswaldo Cruz Institute, Rio de Janeiro, Brazil between June 2006 and November 2007.

Ethical approval was granted for the external validation of the scale and the assessment of inter-observer agreement by the Ethics committee of the London School of Hygiene and Tropical Medicine, the Bangladesh Medical Research Council and the Institutional Review Board of the Oswaldo Cruz Institute.

Patients attending the centres with evidence of a Type 1 reaction or nerve function impairment of less than 6 months duration were eligible. Eligible individuals were invited to participate by the attending physician.

Written informed consent was obtained from individuals who participated in the external validation of the scale and also from those enrolled in the study of inter-observer agreement.

Individuals were examined independently by a worker who was trained to use the scale and experienced leprologists (>20 years experience) who categorized the reaction as mild or moderate or severe. Neither assessor (nor the patient) was aware of the result of the others examination. All of the demographic and clinical data were recorded on a standard form. The Ridley-Jopling classification was used to classify the type of leprosy each patient had [2].

Inter-observer agreement was tested at the two centres in a subsequent stage of the study using the same eligibility criteria. Two assessors independently used the scale to assess individuals diagnosed as having Type 1 reactions. The scale was applied in the same way as in the validation part of the study. The time interval between the two assessments was kept as short as was practicable. Four pairs of assessors were used.

The results were entered into an Access database. The data were analysed using the Statistical Package for the Social Sciences (SPSS version 14. SPSS Inc, Illinois, Chicago).

### Statistical Methods

The item to total score correlation was examined using Spearman rank correlation.

The internal consistency or reliability was assessed using Cronbach's alpha. An alpha between 0.7 and 0.9 is considered acceptable [13]. The contribution of each item in the scale was assessed by calculating Cronbach's alpha for the scale if that item were removed.

The ability of the scale to discriminate between different clinical severity categories was determined using analysis of variance. The threshold for accepting statistical significance was p<0.05.

Inter-observer reliability was evaluated using Intra-Class Correlation of the total score of each examiner using a two-way analysis of variation ( 5% level of significance) and the strength of agreement criteria of Landis and Koch [14]. A Bland Altman plot of the difference between pairs of observations and the mean of those pairs was used to highlight any potential systematic differences between raters.

Receiver Operating Characteristic (ROC) curves were used to determine cut off points for mild, moderate and severe reactions by calculating the sensitivity and specificity of the scale scores for mild and moderate groups and moderate and severe groups respectively.

## Results

### Expert opinion

The questionnaire sent to eight leprologists was returned by seven. The features of Type 1 reaction that were considered important indicators of severity were extent and degree of inflammation of skin lesions, the presence of peripheral oedema, nerve tenderness and nerve function impairment. These parameters are all part of the clinical severity scale we have developed and thus gives our scale face validity.

### Scale testing

81 individuals were recruited (56 from Bangladesh and 25 from Brazil). 64 (79%) were male and 17 (21%) female. The clinical features are summarised in Table 1.

**Table 1:** Characteristics of the participants in each stage of the study, validity and interobserver agreement.

	Validity Number (%)		Interobserver Agreement Number (%)	
**Number**	81		39	
**Gender**	Male 64 (79)		Male 29 (74.4)	
	Female 17 (21)		Female 10 (25.6)	
**Mean Age in years (range)**	39.5 (11–86)		40.9 (11–95)	
**Type of leprosy**	BT	56(69.1)	BT	17 (43.6)
	BB	6 (7.4)	BB	3 (7.7)
	BL	18 (22.2)	BL	15 (38.5)
	LL	4 (4.9)	LL	4 (10.3)
	PNL	1 (1.2)	PNL	0 (0)
**First episode of Type 1 reaction**	52 (64.2)		19 (48.7)	
**Type of reaction**	Skin and nerves	56 (69.1)	Skin and nerves	28 (71.8)
	Skin only	18 (22.2)	Skin only	9 (23.1)
	Nerves only	7 (8.6)	Nerves only	7 (5.1)

The range of the item to total score correlation was −0.09 to +0.73. Nerve pain and nerve tenderness appeared to show no correlation with the total score.

The internal consistency of the scale was assessed using Cronbach's alpha. The Cronbach's alpha was 0.819. Removal of the following individual items resulted in an increase in the alpha: the degree of inflammation of skin lesions, the number of raised inflamed lesions, nerve pain, nerve tenderness, fever, function of right trigeminal nerve, function of the left trigeminal nerve, motor function of the right and left radial nerves (Table 2). This indicates that removal of one or more of these items might improve the ability of the remaining items to measure the severity of Type 1 reactions.

**Table 2:** The Cronbach α for the scale when individual item indicated is removed.

Type of Parameter	Item	Cronbach's Alpha if Item Deleted
**Skin and oedema signs**	*Degree of inflammation of skin*	.*822*
	*Number of raised and/or inflamed lesions*	.*824*
	Peripheral oedema due to reaction	.814
**Nerve symptom**	*Nerve pain and/or paraesthesia*	.*826*
**Nerve sign**	*Nerve tenderness (worst affected nerve only)*	.*825*
**Systemic sign**	*Fever (°C)*	.*820*
**Sensory function of nerve**	*Right trigeminal*	.*821*
	*Left trigeminal*	.*821*
	Right ulnar	.799
	Left ulnar	.789
	Right median	.795
	Left median	.803
	Right posterior tibial	.797
	Left posterior tibial	.800
**Motor function of nerve**	Right facial	.817
	Left facial	.816
	Right ulnar	.810
	Left ulnar	.807
	Right median	.809
	Left median	.808
	*Right radial*	.*821*
	*Left radial*	.*821*
	Right lateral popliteal	.809
	Left lateral popliteal	.816

An increase in α indicates that removal of the item is improving agreement of the remaining scale items. (The overall α for the original 24 item scale was 0.819.)

Principal component analysis (PCA) identified a general factor to which all but nerve pain, nerve tenderness and the number of inflamed lesions contributed accounting for 23.5% of total variance. The important variables in the second factor accounting for 11.6% of the total variance were those related to the eye, namely, trigeminal nerve sensation and facial nerve motor function. The third factor which accounted for 10.7% contrasted individuals with skin signs and no NFI with those who only had NFI.

The severity of the Type 1 reaction was categorized as mild in 19 (23.5%), moderate in 40 (49.4%) and severe in 12 (14.8%). The severity was not recorded in 10 cases.

**Figure 2:**
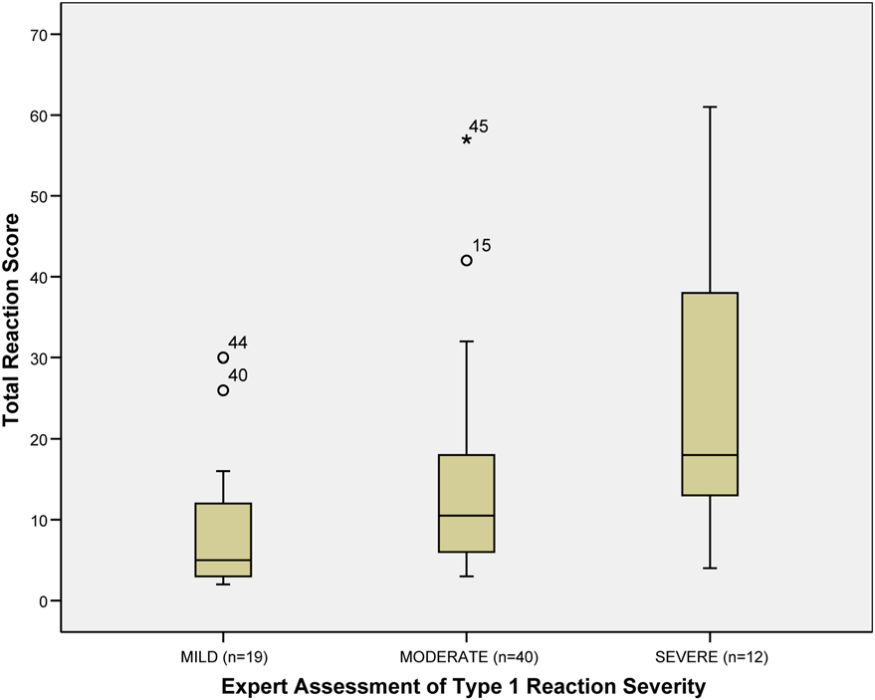
Box plot of the Original Scale Scores by expert severity classification showing medians, interquartile ranges and minimum and maximum scores.

The median scores for each category of reaction severity are shown in the box plots in Fig. 2 with the inter-quartile range (IQR). The median scores for each category were: mild = 5.0 (IQR = 11), moderate = 10.5 (IQR = 13) and severe = 18.0 (IQR = 29).

The differences between the mild and moderate group and the moderate and severe groups did not reach statistical significance (p = 0.053 and 0.052 respectively). The performance of the scale was not materially affected by excluding the seven individuals who did not have skin involvement.

Thirty nine individuals (27 from Bangladesh and 12 from Brazil) were recruited to the second stage of the study to assess inter-observer agreement. The details of these patients are presented in Table 1.

The Intra-Class Correlation coefficient based on a two-way analysis of variance with a random effects model is 0.994. The strength of agreement is very good [14].

**Figure 3:**
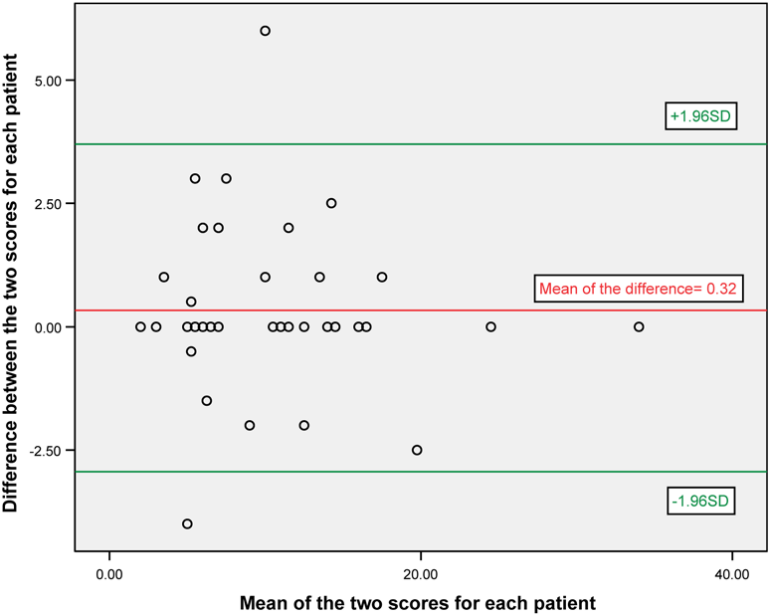
A Bland Altman plot of the difference between the scores of the examiners and the mean of those scores (n = 39).

A Bland and Altman plot [15] (Fig. 3) of the difference between the scores for pairs of observers plotted against the mean of the scores shows good agreement between observers with 95% of differences less than two standard deviations from the mean.

### The Final scale

The scale was adjusted and the analysis repeated in the light of the data obtained (see Appendix S2).

The items nerve pain, nerve tenderness and fever were removed. The rationale for removing these items was that nerve pain and nerve tenderness performed least well of all the items in the scale (in terms of Cronbach's alpha). Fever was removed because occurred in only four of the 120 participants in the study as a whole.

We felt it was important to retain the cutaneous signs and trigeminal and radial nerve function parameters as these are important clinical features of Type 1 reactions.

The scores for the sensory testing (using SWM and cotton wool) were reduced by 50% to make the maximum score possible for each sensory nerve three. This is the maximum score possible for each of the motor and cutaneous items.

These adjustments result in the final scale which consists of 21 items and has a range of 0–63. The maximum score possible for sections A, B and C are 9, 24 and 30 respectively.

For this adjusted version of the scale Cronbach's alpha remained satisfactory at 0.833.

The median scores for each severity group were: mild = 5.0, moderate = 7.5 and severe = 15.25. The differences between the mild and moderate groups (p = 0.038) and the moderate and severe groups (p = 0.048) reached statistical significance.

**Figure 4:**
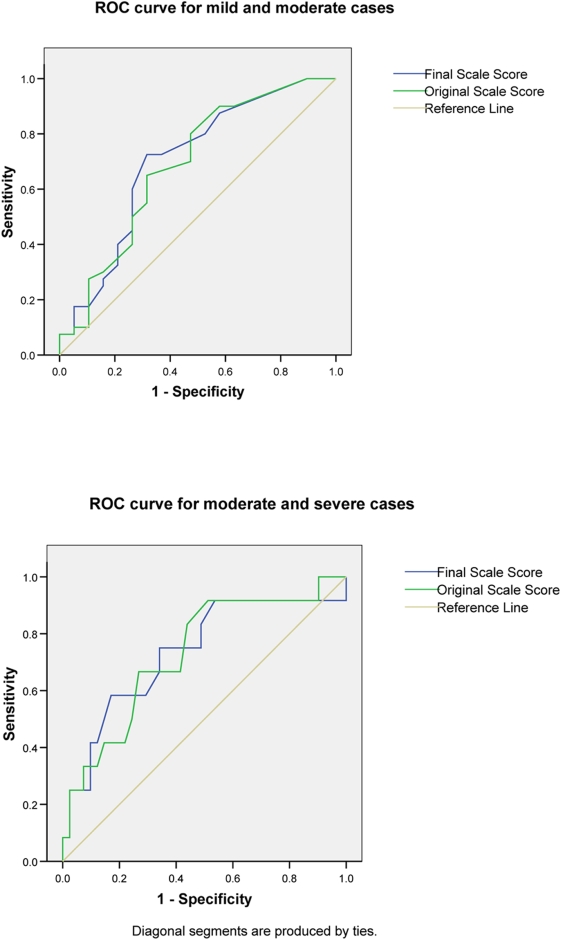
ROC curve for mild and moderate cases and moderate and severe cases.

The ROC curve for the final scale scores was plotted for individuals identified as mild or moderate by the expert raters and for those categorized as moderate or severe (Fig. 4). This facilitates the determination of cut off scores for each category [13].

Using the ROC curves in conjunction with a consideration of the clinical meaning of a given score we determined the following cut off points. A mild Type 1 reaction is characterized using the final scale by a score of 4 or less. A moderate reaction is a score of between 4.5 and 8.5. A severe reaction is a score of 9 or more. The area under the curve for mild and moderate categories is 0.701 for the final scale (0.688 for the original scale). The area under the curve for the moderate and severe categories is 0.734 for the final scale (0.731 for the original scale). These values indicate that the final scale is a fair discriminator between the severity categories traditionally used by clinicians.

## Discussion

In many branches of medicine a single test or diagnostic criterion is either not available or insufficient to adequately measure or describe a clinical syndrome. This has led to difficulties in measuring the severity and prognosis of conditions. The response by researchers has been to develop composite measurement scales.

Psychologists have for many years been concerned with accurately measuring and predicting behaviour and there is a large literature on how to develop and test such measures [13],[16].

The use of unpublished scales to measure outcome has been shown to be a significant source of bias in psychiatry [17]. The lack of clear descriptions of scales and familiarity with them make clinical research difficult to interpret.

We have developed and prospectively validated a reliable 21 item severity scale to measure leprosy Type 1 reactions.

This scale requires the examiner to be proficient in recognising the cutaneous signs of Type 1 reaction, the assessment of VMT and the use of SWM. These skills are not widely practised in many leprosy endemic countries and we anticipate that the main use of this tool, at least initially, will be in the context of research and referral settings.

We believe the scale is easy to use and requires little additional training or equipment for workers based in referral centres. Using a standard assessment form the additional time required to use the scale is minimal.

Type 1 reactions are a significant cause of nerve function impairment and this is the major concern of the physician managing a patient with this condition. The scale we have developed reflects the importance of NFI in the severity of Type 1 reactions.

VMT and SWM in the assessment of NFI have been shown to be reliable [18]. Monofilaments have been shown to be concordant with other sensory function tests [19]. These factors undoubtedly contribute to the robustness of the current scale but careful training and assessment of examiners is required [20].

The use of two monofilaments on the hands (2g and 10g) and feet (10g and 300g) simplifies the system used in the INFIR Cohort Study. However this also results in a higher sensory threshold before an individual's NFI impacts on their Type 1 reaction severity scale score.

The INFIR Cohort study also used a single monofilament test site for the purely sensory radial cutaneous and sural nerves [4]. These two nerves are not commonly tested in routine clinical practice and are not included in the severity scale.

The radial cutaneous and sural nerves may be assessed using various forms of quantitative sensory testing before new impairment identified by monofilaments is demonstrable. Recently published data analysing 188 individuals from the INFIR Cohort who did not present with reaction or nerve involvement has shown that impairment identified using monofilaments occurred in the radial cutaneous nerve in 7% of individuals and in the sural nerve in 6.1% [21]. However the definition of impairment in the radial cutaneous nerve was the inability to feel monofilaments less than 10g or in the sural nerve less than 300g [4].

The lack of a gold standard measure of Type 1 reactions has resulted in us having to compare the scale with the variable and somewhat vague clinical categories of severity as mild, moderate or severe. This has undoubtedly led to a degree of heterogeneity of Type 1 reaction severity within these categories but despite this the scale has performed well.

The final scale has a high degree of inter-observer reliability. We were unable to test intra-observer reliability because of the effect of treatment on the signs of reaction. It would be unethical to withhold treatment. The assessment of intra-observer variation is desirable but not absolutely necessary in scales with a high level of inter-observer reliability [13]. The assessment of intra-observer variation has not been possible in the development of valid scales in other fields such as neurology [22].

In its present form we have found the adjusted scale to be valid and sensitive. Neurological parameters are well represented and reflect the importance of nerve function impairment. The addition of weighting of the different components of the scale would add to its complexity.

A consideration we have not addressed is the performance of the scale in individuals who have nerve damage of greater than 6 months duration. The treatment of nerve damage present for this length of time with corticosteroids is not associated with significant clinical benefit compared to placebo [23]. Nerve damage greater than six months duration should not be included in the severity score. The issue of longstanding NFI can be problematic as patients who are presenting for the first time may be unsure as to the duration of the NFI and may have some acute NFI in a nerve which already has some pre-existing permanent impairment.

Longstanding nerve damage in an individual who experiences a Type 1 reaction would lead to a higher score than an individual with an identical reaction but who has no pre-existing nerve damage. The severity of the Type 1 reaction in the two individuals is presumably the same. However it could be argued that individuals who already have some degree of permanent nerve damage have less neurological reserve and are thus more at risk from even a mild reaction. This however needs to be formally tested.

The scale is currently being used as an additional measure in a clinical trial of methylprednisolone in Type 1 reactions. In this cohort the performance of the scale over time and its ability to reflect change will be assessed.

This is the first prospective validation of a severity scale for leprosy Type 1 reactions. We believe it will prove a useful tool in more accurately assessing Type 1 reactions particularly in clinical trials where the ability to accurately compare the severity of Type 1 reactions in different patients is vital.

## Supporting Information

Appendix S1 (0.08 MB DOC)

Appendix S2 (0.07 MB DOC)
