# Supplementary material for: Development and Validation of a Severity Scale for Leprosy Type 1 Reactions
Source: PLoS Negl Trop Dis. 2008 Dec 23;2(12):e351. doi: 10.1371/journal.pntd.0000351 (PMC2596969; doi:10.1371/journal.pntd.0000351)
Supplement: Appendix S1 — (0.08 MB DOC) [file pntd.0000351.s001.doc]

**APPENDIX S1** – Original Scale

|  | | | **Criteria** | | | | **0** | | **1** | | **2** | | | **3** | | **Score** |  |
| --- | --- | --- | --- | --- | --- | --- | --- | --- | --- | --- | --- | --- | --- | --- | --- | --- | --- |
| A1 | | | Degree of inflammation of skin lesions | | | | None | | **Erythema** | | **Erythema, raised** | | | **Ulceration** | |  |  |
| A2 | | | Number of raised and/or inflamed lesions | | | | **0** | | **1-5** | | **6-10** | | | **>10** | |  |  |
| A3 | | | Peripheral oedema due to reaction | | | | **None** | | **Minimal** | | **Visible, but not affecting function** | | | **Oedema affecting function** | |  |  |
| A4 | | | Nerve pain and/or paraesthesia | | | | **None** | | **Pain on activity** | | **Pain at rest** | | | **Pain disturbing sleep** | |  |  |
| A5 | | | Nerve tenderness (worst affected nerve only) | | | | **None** | | **Mild tenderness** | | **Withdrawal or wincing** | | | **Not allowing palpation** | |  |  |
| A6 | | | Fever (°C) | | | | **<37.5** | | **37.5-38.5** | | **38.6-40** | | | **>40** | |  |  |
| **A SCORE** | | | | | | | | | | | | | | | |  |  |
|  |  | **HANDS** | | **Purple 2g Monofilament scores** | | | | | | **Orange 10g Monofilament scores** | | | | | **Score** | | |
|  |  | **Nerves** | | **0** | **1** | **2** | | **3** | | **4** | | **5** | **6** | |  |  |  |
|  | B1 | RIGHT Trigeminal | | Felt |  | | | | | | | | **Not felt** | |  | | |
|  | B2 | LEFT Trigeminal | | Felt |  |  |  |  |  |  |  |  | **Not felt** | |  | | |
|  | B3 | RIGHT ulnar | | **All sites felt** | **1 site not felt** | **2 sites not felt** | | **3 sites not felt** | | **1 site not felt** | | **2 sites not felt** | **3 sites not felt** | |  | | |
|  | B4 | LEFT ulnar | | **All sites felt** | **1 site not felt** | **2 sites not felt** | | **3 sites not felt** | | **1 site not felt** | | **2 sites not felt** | **3 sites not felt** | |  | | |
|  | B5 | RIGHT median | | **All sites felt** | **1 site not felt** | **2 sites not felt** | | **3 sites not felt** | | **1 site not felt** | | **2 sites not felt** | **3 sites not felt** | |  | | |
|  | B6 | LEFT median | | **All sites felt** | **1 site not felt** | **2 sites not felt** | | **3 sites not felt** | | **1 site not felt** | | **2 sites not felt** | **3 sites not felt** | |  | | |
|  |  | FEET | | **Orange 10g Monofilament scores** | | | | | | **Pink 300g Monofilament scores** | | | | | **Score** | | |
|  |  | Nerves | | **0** | **1** | **2** | | **3** | | **4** | | **5** | **6** | |  |  |  |
|  | B7 | RIGHT posterior tibial | | **All sites felt** | **1 site not felt** | **2 sites not felt** | | **3 sites not felt** | | **1 site not felt** | | **2 sites not felt** | **3 sites not felt** | |  | | |
|  | B8 | LEFT posterior tibial | | **All sites felt** | **1 site not felt** | **2 sites not felt** | | **3 sites not felt** | | **1 site not felt** | | **2 sites not felt** | **3 sites not felt** | |  | | |
|  | **B SCORE** | | | | | | | | | | | | | |  | | |

|  | **NERVE** | **0** | **1** | **2** | **3** | **Score** |
| --- | --- | --- | --- | --- | --- | --- |
| C1 | RIGHT Facial | MRC =5 | **MRC=4** | **MRC=3** | **MRC<3** |  |
| C2 | LEFT Facial | MRC =5 | **MRC=4** | **MRC=3** | **MRC<3** |  |
| C3 | RIGHT Ulnar | MRC =5 | **MRC=4** | **MRC=3** | **MRC<3** |  |
| C4 | LEFT Ulnar | MRC =5 | **MRC=4** | **MRC=3** | **MRC<3** |  |
| C5 | RIGHT Median | MRC =5 | **MRC=4** | **MRC=3** | **MRC<3** |  |
| C6 | LEFT Median | MRC =5 | **MRC=4** | **MRC=3** | **MRC<3** |  |
| C7 | RIGHT Radial | MRC =5 | **MRC=4** | **MRC=3** | **MRC<3** |  |
| C8 | LEFT Radial | MRC =5 | **MRC=4** | **MRC=3** | **MRC<3** |  |
| C9 | RIGHT Lateral Popliteal | MRC =5 | **MRC=4** | **MRC=3** | **MRC<3** |  |
| C10 | LEFT Lateral Popliteal | MRC =5 | **MRC=4** | **MRC=3** | **MRC<3** |  |
| **C SCORE** | | | | | |  |
