# Supplementary material for: Development and Validation of a Severity Scale for Leprosy Type 1 Reactions
Source: PLoS Negl Trop Dis. 2008 Dec 23;2(12):e351. doi: 10.1371/journal.pntd.0000351 (PMC2596969; doi:10.1371/journal.pntd.0000351)
Supplement: Appendix S2 — (0.07 MB DOC) [file pntd.0000351.s002.doc]

APPENDIX S2 – The Final Scale

|  | **Criteria** | **0** | **1** | **2** | **3** | **Score** |
| --- | --- | --- | --- | --- | --- | --- |
| A1 | Degree of inflammation of skin lesions | None | **Erythema** | **Erythema and raised** | **Ulceration** |  |
| A2 | Number of raised and/or inflamed lesions | **0** | **1-5** | **6-10** | **>10** |  |
| A3 | Peripheral oedema due to reaction | **None** | **Minimal** | **Visible, but not affecting function** | **Oedema affecting function** |  |
| **A SCORE** | | | | | |  |

|  | **HANDS** | **Purple 2g Monofilament scores** | | | | **Orange 10g Monofilament scores** | | | **Score** |
| --- | --- | --- | --- | --- | --- | --- | --- | --- | --- |
|  | **Nerves** | **0** | **0.5** | **1** | **1.5** | **2** | **2.5** | **3** |  |
| B1 | RIGHT Trigeminal | Felt |  | | | | | **Not felt** |  |
| B2 | LEFT Trigeminal | Felt |  |  |  |  |  | **Not felt** |  |
| B3 | RIGHT ulnar | **All sites felt** | **1 site not felt** | **2 sites not felt** | **3 sites not felt** | **1 site not felt** | **2 sites not felt** | **3 sites not felt** |  |
| B4 | LEFT ulnar | **All sites felt** | **1 site not felt** | **2 sites not felt** | **3 sites not felt** | **1 site not felt** | **2 sites not felt** | **3 sites not felt** |  |
| B5 | RIGHT median | **All sites felt** | **1 site not felt** | **2 sites not felt** | **3 sites not felt** | **1 site not felt** | **2 sites not felt** | **3 sites not felt** |  |
| B6 | LEFT median | **All sites felt** | **1 site not felt** | **2 sites not felt** | **3 sites not felt** | **1 site not felt** | **2 sites not felt** | **3 sites not felt** |  |
|  | FEET | **Orange 10g Monofilament scores** | | | | **Pink 300g Monofilament scores** | | | **Score** |
|  | Nerves | **0** | **0.5** | **1** | **1.5** | **2** | **2.5** | **3** |  |
| B7 | RIGHT posterior tibial | **All sites felt** | **1 site not felt** | **2 sites not felt** | **3 sites not felt** | **1 site not felt** | **2 sites not felt** | **3 sites not felt** |  |
| B8 | LEFT posterior tibial | **All sites felt** | **1 site not felt** | **2 sites not felt** | **3 sites not felt** | **1 site not felt** | **2 sites not felt** | **3 sites not felt** |  |
| **B SCORE** | | | | | | | | |  |

|  | **NERVE** | **0** | **1** | **2** | **3** | **Score** |
| --- | --- | --- | --- | --- | --- | --- |
| C1 | RIGHT Facial | MRC =5 | **MRC=4** | **MRC=3** | **MRC<3** |  |
| C2 | LEFT Facial | MRC =5 | **MRC=4** | **MRC=3** | **MRC<3** |  |
| C3 | RIGHT Ulnar | MRC =5 | **MRC=4** | **MRC=3** | **MRC<3** |  |
| C4 | LEFT Ulnar | MRC =5 | **MRC=4** | **MRC=3** | **MRC<3** |  |
| C5 | RIGHT Median | MRC =5 | **MRC=4** | **MRC=3** | **MRC<3** |  |
| C6 | LEFT Median | MRC =5 | **MRC=4** | **MRC=3** | **MRC<3** |  |
| C7 | RIGHT Radial | MRC =5 | **MRC=4** | **MRC=3** | **MRC<3** |  |
| C8 | LEFT Radial | MRC =5 | **MRC=4** | **MRC=3** | **MRC<3** |  |
| C9 | RIGHT Lateral Popliteal | MRC =5 | **MRC=4** | **MRC=3** | **MRC<3** |  |
| C10 | LEFT Lateral Popliteal | MRC =5 | **MRC=4** | **MRC=3** | **MRC<3** |  |
| **C SCORE** | | | | | |  |

| **Total score** | **Scores of A+B+C** |  |
| --- | --- | --- |
